# Pharmacological Actions, Molecular Mechanisms, Pharmacokinetic Progressions, and Clinical Applications of Hydroxysafflor Yellow A in Antidiabetic Research

**DOI:** 10.1155/2021/4560012

**Published:** 2021-12-13

**Authors:** Xilan Zhang, Dayue Shen, Yating Feng, Yuanping Li, Hui Liao

**Affiliations:** ^1^School of Pharmacy, Shanxi Medical University, Taiyuan 030001, China; ^2^Department of Pharmacy, Fifth Hospital of Shanxi Medical University (Shanxi Provincial People's Hospital), Taiyuan 030012, China

## Abstract

Hydroxysafflor yellow A (HSYA), a nutraceutical compound derived from safflower (*Carthamus tinctorius*), has been shown as an effective therapeutic agent in cardiovascular diseases, cancer, and diabetes. Our previous study showed that the effect of HSYA on high-glucose-induced podocyte injury is related to its anti-inflammatory activities via macrophage polarization. Based on the information provided on PubMed, Scopus and Wanfang database, we currently aim to provide an updated overview of the role of HSYA in antidiabetic research from the following points: pharmacological actions, molecular mechanisms, pharmacokinetic progressions, and clinical applications. The pharmacokinetic research of HSYA has laid foundations for the clinical applications of HSYA injection in diabetic nephropathy, diabetic retinopathy, and diabetic neuropathy. The application of HSYA as an antidiabetic oral medicament has been investigated based on its recent oral delivery system research. *In vivo* and *in vitro* pharmacological research indicated that the antidiabetic activities of HSYA were based mainly on its antioxidant and anti-inflammatory mechanisms via JNK/c-jun pathway, NOX4 pathway, and macrophage differentiation. Further anti-inflammatory exploration related to NF-*κ*B signaling, MAPK pathway, and PI3K/Akt/mTOR pathway might deserve attention in the future. The anti-inflammatory activities of HSYA related to diabetes and diabetic complications will be a highlight in our following research.

## 1. Introduction

**Figure 1:**
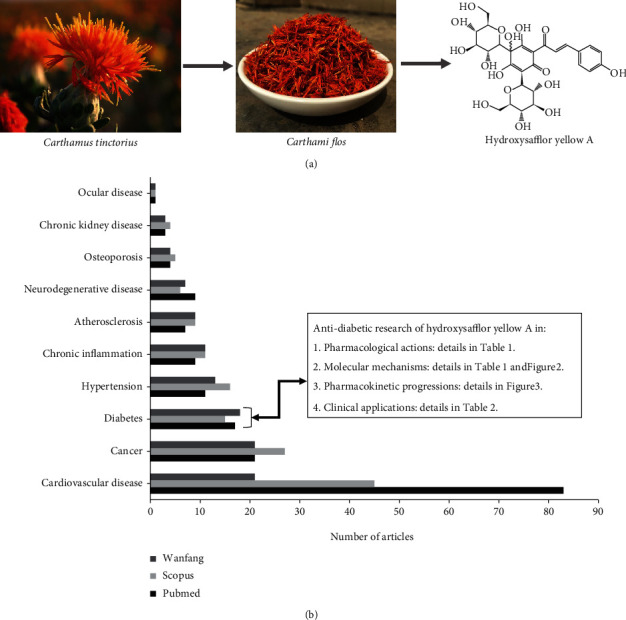
The source, structure, and literature research of hydroxysafflor yellow A: (a) the source and structure of hydroxysafflor yellow A; (b) the research articles of the top ten diseases related to hydroxysafflor yellow A. Abbreviation: Wanfang: Wanfang Data.

Hydroxysafflor yellow A (HSYA) is a single chalcone glycoside compound [1] which is derived from safflower (*Carthamus tinctorius*), a traditional Chinese herb (Figure 1) [2]. The most general and traditional method of extracting HSYA is water immersion. However, many other extraction systems have been developed such as smashing tissue extraction, microwave extraction, ultrasound extraction, and Soxhlet extraction [3]. HSYA has been commonly used in China to treat cardiovascular disease (CVD) [4]. Our recent literature research provides a number of articles and reviews describing novel applications of HSYA towards various diseases such as cancer and diabetes, beyond their conventional use against CVD (Figure 1).

According to the International Diabetes Federation, the number of adults diagnosed with diabetes has increased from 285 million in the year 2009 to 463 million in the year 2019, 95% of which are type 2 diabetes mellitus (T2DM) patients [5]. Diabetes is associated with accelerated rates of macrovascular and microvascular complications [6]. Macrovascular complications affect the heart, brain, and peripheral arteries and are termed CVD, cerebrovascular disease, and peripheral vascular disease, respectively [7]. Diabetes-related macrovascular complications are responsible for the impaired quality of life, disability, and premature death associated with diabetes [8, 9]. Microvascular complications affect the retina which is the inner part of the eye, the kidneys, and peripheral nerves. The resulting conditions are known as diabetic retinopathy, diabetic nephropathy (DN), and diabetic neuropathy, respectively [7]. In a study involving 689 individuals with T2DM obtained at baseline, the occurrences of microvascular complications observed during a median follow-up of 10.5 years were as follows: 206 patients had DN, 161 patients had retinopathy, and 179 patients had neuropathy [10].

Specifically, T2DM is characterized by chronic systemic inflammation alongside hyperglycemia and insulin resistance in the body [11]. Clinical data analysis showed that elevated C-reactive protein (CRP), tumor necrosis factor- (TNF-) *α*, and interleukin- (IL-) 6 were the most common inflammation indicators in diabetes-related angiopathies [12]. Related research suggested that HSYA could inhibit the apoptosis of pancreatic *β*-cells, and this might be the underlying mechanisms through which HSYA regulates glycolipid metabolism in T2DM rats [13]. Our previous study indicated that HSYA had direct protective effects on high glucose- (HG-) induced podocyte injury and indirect protective effects by regulating macrophage M1/M2 polarization [14]. These effects were related to its antioxidant and anti-inflammatory activities *in vitro* [15].

In this article, we first reviewed the *in vivo* and *in vitro* antidiabetic pharmacological actions and antidiabetic molecular mechanisms of HSYA. Based on signal research in the application of HSYA in the treatment of inflammation-related diseases, possible anti-inflammatory pathways involved in antidiabetic effects were discussed. The clinical applications of HSYA in diabetic macrovascular and microvascular complications were then summarized based on its recent pharmacokinetic progression. Finally, possible application of HSYA as an antidiabetic oral medicament was investigated.

## 2. Antidiabetic Pharmacological Research and Related Mechanisms

### 2.1. *In Vivo* Antidiabetic Research

T2DM was induced in rats by feeding high-fat diet (HFD) for four weeks followed by intraperitoneal injection of streptozocin (STZ). The established models were treated with HSYA for eight weeks while metformin was used as positive control. The results showed that the underlying mechanisms of HSYA in T2DM rats were related to the following activities: the direct or indirect inhibition of pancreatic *β*-cell apoptosis, the improvement of insulin resistance, and the regulation of glycolipid metabolism [13].

Also, in the treatment of HFD- and STZ-induced T2DM rats by HSYA, renal protective effects were observed based on the improvement of renal functions including serum creatinine (Scr), blood urea nitrogen (BUN), glomerular volume, podocyte number, and cell apoptosis markers. Furthermore, in the HSYA treatment group, the levels of TNF-*α* and the inflammatory products, including free fatty acids (FFA) and lactic dehydrogenase (LDH), were significantly decreased. Regarding oxidative stress markers, the level of superoxide dismutase (SOD) markedly increased in the HSYA treatment group, while the level of malondialdehyde (MDA) in the serum and kidney tissue evidently decreased [16].

In STZ-induced type 1 diabetes mellitus (T1DM) rats, a diabetic wound model was established by full-thickness excisional wounds that extended through the panniculus carnosus with a biopsy punch. Topical application of HSYA significantly enhanced the wound closure rate, and the time taken for complete wound closure was 17 days, whereas 30 days was needed for complete wound closure with phosphate-buffered saline (PBS) treatment [17].

### 2.2. *In Vitro* Antidiabetic Research

*In vitro* antidiabetic studies were conducted on seven different cell lines: rat INS-1 insulinoma cells [18], mice MPC-5 podocyte cells [14], human umbilical vein endothelial cells (HUVECs) [17, 19], human brain microvascular endothelial cells (HBMECs) [20], 3T3-L1 preadipocytes and adipocytes [21], and RAW264.7 macrophage cells [14, 17].

The loss of functional insulin-producing *β*-cells is a hallmark of diabetes; therefore, understanding the cellular biology of the pancreas is crucial. Rat insulinoma INS-1 cells are widely used to study glucose-stimulated insulin secretion [22]. DN is one of the microvascular complications of diabetes and is a main cause of end-stage nephropathy. The most common clinical feature of DN is progressive proteinuria which is related to podocyte function. Podocyte plays an important role in maintaining the integrity and function of the glomerular filtration barrier. MPC-5 cell line is also widely used to evaluate renal injury *in vitro* [23]. Related research showed that HG-induced apoptosis of podocytes and pancreatic *β*-cells was reversed by HSYA [14, 18].

In diabetic patients, hyperglycemia-induced endothelial injury results in all kinds of vascular complications [24]. *In vitro* research showed that HG increased HUVEC apoptosis, vascular permeability, monocyte adhesion, the formation of reactive oxygen species (ROS), and the expression of NADPH oxidase 4 (NOX4) protein. The increased vascular injury by HG was attenuated by HSYA [19]. Another *in vitro* research showed that HSYA could inhibit methylglyoxal-induced injury in cultured HBMEC, which was associated with its antiglycation effect. Methylglyoxal is mainly formed from the degradation of glucose and glycated proteins [20].

For both diabetic wounds and DN progression, a central feature is the persistence of chronic inflammation, which is partly due to the prolonged presence of proinflammatory macrophages [25, 26]. In HG- and lipopolysaccharide- (LPS-) induced RAW264.7 macrophage cells, HSYA showed its anti-inflammatory effects by decreasing TNF-*α*, IL-1*β*, and nitric oxide (NO) levels [14, 17]. From Table 1, we could see that the main antidiabetic mechanism of HSYA is through its anti-inflammatory activity.

### 2.3. Anti-Inflammatory Signals in Antidiabetic Research of HSYA

So far, our review has shown that the antidiabetic mechanisms of HSYA are related to the following signals: c-jun NH2-terminal kinases/c-jun (JNK/c-jun) pathway [18], NOX4 pathway [19], macrophage polarization [14], and phosphoinositide 3-kinase/protein kinase B (PI3K/Akt) pathway [13]. HSYA also showed its ability to cause a decrease in oxidative stress factors such as ROS [18, 19] and hydrogen peroxide (H_2_O_2_) [19].

Inflammation is closely linked to the pathogenesis of diabetes, and chronic inflammation is one of the main causes of insulin resistance. Proinflammatory mediators can be related to obesity and induce insulin resistance in adipose tissue. Signaling pathways of transcription factors, particularly nuclear factor-*κ*B (NF-*κ*B) signaling, are involved in insulin sensitivity [27]. NF-*κ*B plays a crucial role in the development of diabetic complications because of its involvement in the expression of genes that are responsible for the damage of organs such as the brain, liver, heart, muscles, endothelium, adipose tissue, and pancreas by inflammation, apoptosis, and oxidative stress [28].

**Figure 2:**
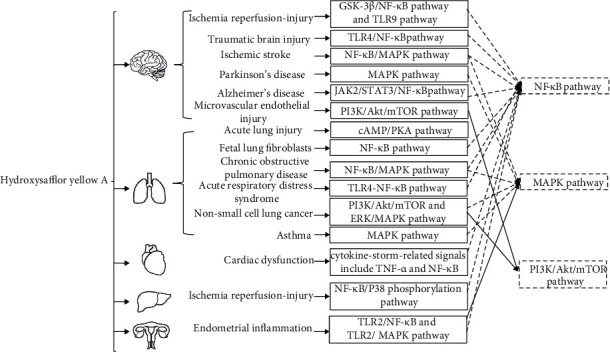
The molecular mechanisms of hydroxysafflor yellow A in the treatment of inflammation-related diseases. Abbreviations: GSK3*β*: glycogen synthase kinase-3*β*; NF-*κ*B: nuclear factor-*κ*B; TLR9: toll-like receptor 9; TLR4: toll-like receptor 4; TLR2: toll-like receptor 2; MAPK: mitogen-activated protein kinase; JAK2: Janus kinase 2; STAT3: signal transducers and activators of transcription 3; PI3K: phosphatidylinositol 3-kinase; Akt: protein kinase B; mTOR: mammalian target of rapamycin; cAMP: cyclic adenosine monophosphate; PKA: protein kinase A; ERK: extracellular signal-regulated kinase; TNF-*α*: tumor necrosis factor-*α*.

The role of NF-*κ*B signal in the antidiabetic activities of HSYA has not been previously reported.  Figure 2 indicates that NF-*κ*B signal plays an important role in the use of HSYA to treat other inflammatory diseases [29–38]. This might provide some research points to explore anti-inflammatory mechanisms of HSYA in the treatment of diabetes and diabetes complications.

### 2.4. Possible Anti-Inflammatory Mechanisms in Antidiabetic Research of HSYA

In Figure 2, organ damages treated by HSYA via the NF-*κ*B pathway include brain damages such as ischemia reperfusion-injury, traumatic brain injury, ischemic stroke, and Alzheimer's disease (AD) [29–32]; lung injury such as fetal lung fibroblasts, chronic obstructive pulmonary disease, and acute respiratory distress syndrome [33–35]; cardiac dysfunction [36]; liver ischemia reperfusion-injury [37]; and endometrial inflammation [38].

It is reported that AD and T2DM share many common features including inflammation, oxidative stress, and neuronal degeneration [39]. *β*-Amyloid- (A*β*-) mediated inflammation plays a critical role in the initiation and progression of AD. HSYA protects A*β*-induced AD model by inhibiting inflammatory response, which may involve inhibiting the activation of the NF-*κ*B pathway [32]. The NF-*κ*B signaling pathway will be the first research point for our team in future studies on the antidiabetic mechanisms of HSYA.

Mitogen-activated protein kinase (MAPK) pathway and phosphoinositide 3-kinase/protein kinase B/mammalian target of rapamycin (PI3K/Akt/mTOR) pathway are two other important signals indicating the intervention of HSYA in the treatment of Parkinson's disease (PD) [40], asthma [41], non-small-cell lung cancer [42], and brain microvascular endothelial injury [43]. It is reported that activated MAPK may be associated with both inflammation and energy metabolism in mice, rats, and humans fed with HFD for a short or long term [44]. A case-control study including 248 cases of T2DM and 101 controls showed that genetic variations in the PI3K/Akt/mTOR signaling pathway may be associated with increasing risk of obesity and diabetes [45]. Our previous discussion showed that HSYA could promote the activation of PI3K/Akt and inhibit the apoptosis of pancreatic *β*-cells in HFD- and STZ-induced T2DM rats [13]. Further research on the relationship between HSYA and PI3K/Akt/mTOR signal in diabetic complications should be conducted.

Other inflammation signals, including toll-like receptor 9 (TLR9) signal and cyclic adenosine monophosphate/protein kinase A (cAMP/PKA) signal, were observed in ischaemic cortex after cerebral ischaemia and reperfusion and acute lung injury [46, 47]. TLRs are a family of pattern recognition receptors that play a critical role in innate immune response. Recently, studies have reported the important role of TLR4 pathway in insulin resistance [27]. TLRs can be proposed as new targets in the intervention of HSYA in diabetes. The important roles of cAMP/PKA signal in the cognitive impairment of diabetic rats may suggest its involvement in the antidiabetic mechanism of HSYA.

## 3. Pharmacokinetic Progressions

### 3.1. Pharmacokinetics of Intravenous Administration

#### 3.1.1. In Healthy Humans

36 healthy volunteers were recruited in a single-center, open-label, single-dose, and multiple-dose study. It was found that the area under the curve (AUC) of plasma concentration at different time points and time to peak plasma concentration (Cmax) were linearly related to the dose ranging from 25 to 75 mg in a single administration of HSYA and the elimination half-life was about 3.91–4.18 h. When HSYA was administered for 7 d (50 mg/d) continuously, Cmax and AUC decreased significantly and the elimination half-life was prolonged from 3.91 h to 4.41 h [48].

Pharmacokinetic studies in healthy humans have shown that the metabolic process in the body after intravenous administration of HSYA conforms to the two-compartment model, indicating that HSYA can be quickly distributed in many organs including the heart, liver, spleen, lungs, brain, intestines, and kidneys [49]. The distribution of HSYA in the kidneys is more than that of the other organs [3]. The excretion of HSYA is mainly from the kidneys, and the cumulative excretion rate of HSYA in urine 24 h after intravenous administration is up to 88.6% [3, 49]. According to the above characteristics, the pharmacokinetic indexes of HSYA in DN patients are different from those of the healthy volunteers.

#### 3.1.2. In Renal Insufficient Patients

It was found that the Cmax and AUC of HSYA in the diabetic impaired renal function group increased and the apparent volume of distribution and clearance rate reduced significantly after a single administration. The results showed that impaired renal function affected pharmacokinetic indicators [50].

Relevant studies have shown that after administering HSYA intravenously for 1 h, the average blood concentration of HSYA in renal insufficient patients was equivalent to 2.64 times that of patients with normal renal functions [51]. It is suggested that the dosage and frequency of administration should be adjusted according to the blood concentration when HSYA is used in DN patients.

#### 3.1.3. In Patients with Traumatic Brain Injury

A sensitive, rapid, and reliable liquid chromatography-tandem mass spectrometry method was applied to investigate the pharmacokinetics of HSYA in patients with traumatic brain injury (TBI). The results demonstrated that some HSYA crossed the blood-brain barrier after administration. This study provides evidence to better understand the pharmacokinetics and potential clinical guidance for TBI treatment [52].

#### 3.1.4. Clinical Antidiabetic Applications

Safflower yellow injection (SYI) containing 90% HSYA (45 mg HSYA per 50 mg SYI) has been widely used clinically [53]. In line with clinical guidelines and expert consensus [54], the use of SYI is becoming more and more standardized. Randomized controlled trials (RCTs) of SYI in the treatment of diabetes complications are summarized in Table 2 [55–61].

It can be observed from Table 2 that HSYA has effects on microvascular complications as well as macrovascular complications. Among these complications, HSYA was mostly used in the early stage of DN, and the mechanism research showed that HSYA had anti-inflammatory activity by decreasing TNF-*α* levels in DN patients [62]. There is no clinical anti-inflammatory research about HSYA on diabetic retinopathy, diabetic neuropathy, etc. The development of further clinical applications of HSYA may need to be carried out alongside its clinical anti-inflammatory effects.

It is said that the age of natural antioxidant compounds in the treatment of diabetic complications is coming [63]. HSYA injection has made some progress in the treatment of different diabetic complications. But from the perspective of patients, it is obvious that an oral drug is more convenient than an injection.

### 3.2. Pharmacokinetics of Oral Administration

#### 3.2.1. In Healthy Humans

The pharmacokinetics of HSYA in 12 healthy volunteers after a single oral administration of HSYA was investigated. The plasma pharmacokinetics of HSYA after oral administration in the 12 healthy subjects showed that the component was absorbed quickly, with a peak time of 1 h and a short elimination half-life of approximately 2.6–3.5 h [64]. HSYA is relatively polar and easily catabolized and metabolized in the gastrointestinal tract and liver, leading to its rapid elimination, short half-life, and low bioavailability under oral or intragastric administration conditions [49]. The clinical use of HSYA as an oral preparation is being hindered by its low bioavailability, and hence, there is the need for an improvement of its oral bioavailability.

#### 3.2.2. Oral Delivery System Research

**Figure 3:**
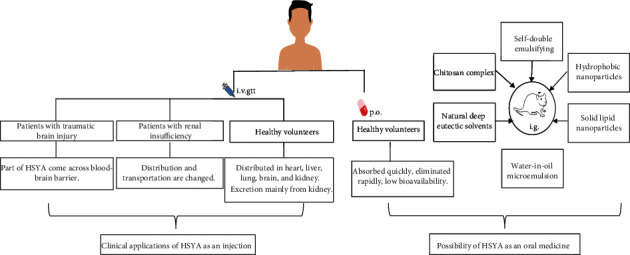
Clinical applications and possible medicine development of HSYA based on human and animal pharmacokinetic research. Abbreviation: HSYA: hydroxysafflor yellow A.

Fortunately, research on the delivery system of HSYA made it possible to develop it into an oral medicament. Some oral delivery systems of HSYA are shown in Figure 3. They are water-in-oil microemulsion [65], self-double-emulsifying [66], hydrophobic nanoparticles [67], chitosan complex [68], solid lipid nanoparticles [69], and natural deep eutectic solvents [70].

Related research suggested that shell nanoparticles are a highly effective delivery system for resveratrol, another natural compound, due to their significant effects in increasing the bioavailability and anti-inflammation activity [71]. We look forward to a suitable delivery system for HSYA, which will improve not only its bioavailability but also its anti-inflammatory activity in the near future.

## 4. Conclusion

HSYA, a major active component from safflower plant, has drawn more interest in recent years for its multiple pharmacological actions. We aim to provide an updated overview of HSYA in diabetes and diabetic complications from these four points: pharmacological actions, molecular mechanisms, pharmacokinetic progressions, and clinical applications. Anti-inflammation mechanism plays an important role in the antidiabetic pharmacological actions of HSYA. Further anti-inflammation research should pay attention to more inflammation signals such as NF-*κ*B pathway and MAPK pathway. The pharmacokinetic properties of HSYA enhanced its wide clinical use as an injection to treat diabetic complications. Based on the development of drug delivery systems, HSYA could be expected as an oral drug with improved bioavailability and improved anti-inflammatory activity.

## Figures and Tables

**Table 1:** Summary of pharmacological effects and mechanisms of HSYA on diabetes and diabetes complications.

Disease	Species/strains	Effective dose/concentration	Route	Positive control	Intervention time	Main improved results	Mechanisms/pathways	Reference
Diabetes	HFD- and STZ-induced T2DM rats	120 mg/kg	i.g.	Metformin as positive control	8 weeks	Pancreatic *β*-cell apoptosis↓, FBG↓, IR↓, TG↓, TC↓, LDLC↓, glycogen synthase↑, hepatic glycogen↑	Regulation on glycolipid metabolism via PI3K/Akt pathway	[13]
Diabetes	HG-induced rat INS-1 insulinoma cells (pancreatic *β*-cells)	800 *μ*M		N-Acetylcysteine as oxidative stress scavenger control	72 hours	Pancreatic *β*-cell apoptosis↓, ROS↓, MDA↓, CAT↑, GSH-px↑, SOD↑	Antioxidative effects via JNK/c-jun pathway	[18]
Diabetic nephropathy	HFD- and STZ-induced T2DM rats	120 mg/kg	i.g.		8 weeks	Scr↓, UN↓, TG↓, TC↓, LDLC↓, FBG↓, TNF-*α*↓, LDH↓, FFA↓, MDA↓, SOD↑	Antioxidative and anti-inflammation effects	[16]
Diabetic nephropathy	HG-induced mice MPC-5 podocyte cells and HG-induced mice RAW264.7 cells	100 *μ*M, 200 *μ*M		Kaempferol as positive control	24 hours	Podocyte apoptosis↓ In podocytes: TNF-*α*↓, IL-1*β*↓ In RAW264.7 cells: TNF-*α*↓, iNOS↓, IL-1*β*↓ CD206↑, Arg-1↑	Anti-inflammation effects directly on podocyte cells and indirectly via macrophage polarization	[14]
Diabetic vascular injury	HG-induced HUVECs	10 *μ*M, 25 *μ*M, 50 *μ*M			24, 48, and 72 hours	HUVEC hyperpermeability↓, HUVEC apoptosis↓, VCAM-1↓, ICAM-1↓, E-selectin↓, NOX4↓, ROS↓, H_2_O_2_↓	Anti-inflammation effects via the NOX4 pathway	[19]
Diabetic vascular injury	Methylglyoxal-induced HBMECs	10, 50, and 100 *μ*M			24 hours	HBMEC apoptosis↓, caspase-3↓, AGEs↓	Antiglycation effects	[20]
Diabetic wound	STZ-induced T1DM rats	2 mg/mL	vs ext	Hydrogel as positive control	30 days	Wound closure↑, granulation tissue formation↑, collagen disposition↑, secretion of VEGF↑, TGF-*β*1↑		[17]
Diabetic wound	HUVECs and LPS-induced RAW264.7 cells	0.4, 0.8, and 1.6 mM			60 and 96 hours	NO production↓, HEK migration↑, HUVEC tube formation↑	Anti-inflammation effects	[17]
Diabetic obesity	3T3-L1 preadipocytes and adipocytes	100 mg/L			24 hours	PPAR*γ*2 promoter activities↑, PPAR*γ*2↑	Increasing the expression of insulin signaling pathway-related genes	[21]

Abbreviations: T1DM: type 1 diabetes mellitus; T2DM: type 2 diabetes mellitus; HFD: high-fat diet; STZ: streptozotocin; FBG: fasting blood glucose; IR: insulin resistance; TG: triglyceride; TC: total cholesterol; LDLC: low-density lipoprotein cholesterol; DN: diabetic nephropathy; ROS: reactive oxygen species; SOD: superoxide dismutase; CAT: catalase; GSH-px: glutathione peroxidase; MDA: malondialdehyde; Scr: serum creatinine; UN: urea nitrogen; LDH: lactate dehydrogenase; FFA: free fatty acids; NOX4: NADPH oxidase 4; H_2_O_2_: hydrogen peroxide; HG: high glucose; HBMECs: human brain microvascular endothelial cells; HUVECs: human umbilical vein endothelial cells; VCAM-1: vascular cell adhesion molecule-1; ICAM-1: intercellular adhesion molecule-1; iNOS: inducible nitric oxide synthase; TNF-*α*: tumor necrosis factor-*α*; CD206: mannose receptor; Arg-1: arginase-1; IL-1*β*: interleukin-1*β*; LPS: lipopolysaccharide; AGEs: advanced glycation end-products; VEGF: vascular growth factors; TGF-*β*1: transforming growth factor-*β*1; NO: nitric oxide; HEKs: human epithelial keratinocytes; PPAR*γ*2: peroxisome proliferator-activated receptor-*γ*2.

**Table 2:** The RCT research of SYI (90% HSYA) in the treatment of diabetes and diabetes complications.

	Diseases	RCT research	Main improved clinical indicators	Mechanism research	Reference
Microvascular complications	Diabetic nephropathy	Early stage: *n* = 535 (control: *n* = 532)	Serum creatinine	SOD, MDA, TNF-*α*, IL-6, and IL-10	[55]
		End stage: *n* = 50 (control: *n* = 50)	24 h proteinuria, urea nitrogen		[56]
	Diabetic retinopathy	*n* = 92 (control: *n* = 76)	Serum vascular endothelial growth factor and endostatin		[57]
	Diabetic neuropathy	*n* = 41 (control: *n* = 41)	Tendon reflexes and EMG nerve conduction velocity		[58]
Macrovascular complications	Cardiovascular disease	Unstable angina pectoris: *n* = 42 (control: *n* = 42)	Number and duration of angina pectoris		[59]
	Cerebrovascular disease	Acute cerebral infarction: *n* = 40 (control: *n* = 40)	NIHSS score		[60]
	Peripheral vascular disease	Diabetic foot ulcers: *n* = 20 (control: *n* = 20)	Wagner classification		[61]

Abbreviations: RCT: randomized controlled trial; SYI: safflower yellow injection; HSYA: hydroxysafflor yellow A; SOD: superoxide dismutase; MDA: malondialdehyde; TNF-*α*: tumor necrosis factor-*α*; IL-6: interleukin-6; IL-10: interleukin-10; EMG: electromyogram; NIHSS: National Institute of Health Stroke Scale.

## Data Availability

All authors declare that the readers can access the conclusions from the three figures and two tables. All of the figures and tables are summarized based on the references.
